# Epigenetics in Lewy Body Diseases: Impact on Gene Expression, Utility as a Biomarker, and Possibilities for Therapy

**DOI:** 10.3390/ijms21134718

**Published:** 2020-07-02

**Authors:** Aintzane Urbizu, Katrin Beyer

**Affiliations:** Department of Pathology, Germans Trias i Pujol Research Institute, 08916 Badalona, Spain; aurbizu@igtp.cat

**Keywords:** Parkinson’s disease, dementia with Lewy bodies, Lewy body diseases, epigenetics, DNA methylation, histone modification, alpha-synuclein

## Abstract

Lewy body disorders (LBD) include Parkinson’s disease (PD) and dementia with Lewy bodies (DLB). They are synucleinopathies with a heterogeneous clinical manifestation. As a cause of neuropathological overlap with other neurodegenerative diseases, the establishment of a correct clinical diagnosis is still challenging, and clinical management may be difficult. The combination of genetic variation and epigenetic changes comprising gene expression-modulating DNA methylation and histone alterations modifies the phenotype, disease course, and susceptibility to disease. In this review, we summarize the results achieved in the deciphering of the LBD epigenome. To provide an appropriate context, first LBD genetics is briefly outlined. Afterwards, a detailed review of epigenetic modifications identified for LBD in human cells, postmortem, and peripheral tissues is provided. We also focus on the difficulty of identifying epigenome-related biomarker candidates and discuss the results obtained so far. Additionally, epigenetic changes as therapeutic targets, as well as different epigenome-based treatments, are revised. The number of studies focusing on PD is relatively limited and practically inexistent for DLB. There is a lack of replication studies, and some results are even contradictory, probably due to differences in sample collection and analytical techniques. In summary, we show the current achievements and directions for future research.

## 1. Introduction

The group of Lewy body disorders (LBD) comprises Parkinson’s disease (PD) and dementia with Lewy bodies (DLB). Whereas PD is one of the most prevalent movement disorders, DLB is after Alzheimer’s disease (AD) as the second most common cause of degenerative dementia. LBD belong to the group of synucleinopathies and are characterized by the abnormal accumulation and deposition of misfolded and aggregated alpha-synuclein (α-syn) giving rise to Lewy bodies and Lewy neurites [1].

The major clinical features of PD include slowness of movement, the decrease of amplitude and speed, as well as bradykinesia, rest tremor, and rigidity. Up to 75% of PD patients develop dementia (PD with dementia; PDD) after 10 years of PD diagnosis [2], almost 85% after 20 years [3], and by the age of 90, around 95% of PD patients present dementia [4].

The recently revised guidelines for the diagnosis and management of DLB define fluctuating cognition with pronounced variations in attention and alertness, recurrent visual hallucinations, REM sleep behavior disorder (RBD), and at least one spontaneous cardinal features of parkinsonism as core clinical features of the disease [5]. Additional clinical features include severe sensitivity to antipsychotic agents, postural instability, repeated falls, and severe autonomic dysfunction.

PD and DLB present hyposmia and RBD as early and mostly preclinical symptoms. Longitudinal studies have shown that patients presenting the idiopathic form of RBD convert to PD or DLB, with an estimated risk of conversion up to 91% after 14 years of follow-up from RBD diagnosis [6]. Correspondingly, it is now accepted that RBD is a manifestation of prodromal PD and DLB [7,8].

As proposed initially by Braak and colleagues, in PD, α-syn deposition starts in the dorsal motor nucleus of the vagus and spreads to the locus coeruleus. Thereafter, still in early disease stages, it affects the brainstem, leading to the loss of dopaminergic neurons in the substantia nigra (SN) pars compacta, and at this phase, PD can be clinically diagnosed [9]. With disease progression, α-syn pathology propagates through the brain following a predominantly caudo-rostral route until affecting the neocortex [10].

On the contrary, in DLB, α-syn pathology is found in cortical areas from the beginning of the disease. To explain the early involvement of the neocortex, a recent hypothesis proposed that in the case of DLB, α-syn pathology propagates via an olfactory route [11]. Accordingly, after the early involvement of the olfactory bulb, α-syn pathology rapidly affects limbic regions and reaches neocortical areas directly after. This route of α-syn progression would explain the development of dementia at early disease stages, at the same time or even before parkinsonian symptoms [11,12].

A pronounced neuropathological overlap between DLB and PDD has been observed. Consequently, it is very difficult to distinguish between brains from patients who died from DLB and those who died from PDD [13]. Although the main pathological finding in DLB and PDD is the widespread distribution of α-syn pathology, an elevated percentage of both also presents concomitant AD pathology [14]. The load of neurofibrillary tangles, together with beta-amyloid pathology, is indicative for the interval between motor symptoms and dementia onset, as well as for patient survival [15]. In PDD, the severity of dementia correlates with the distribution of α-syn pathology and its combination with AD-pathology. More than 50% of all PDD brains show severe stages of both pathology types [16]. The main differences between PDD and DLB include a more severe cell loss in the SN in PDD [17], which corresponds to more advanced parkinsonism in these cases. Moreover, temporal and parietal cortices present a higher burden of α-syn pathology in DLB compared to PDD [18], and hallucinations in DLB do not correlate with the cholinergic deficit in the pedunculopontine nucleus [19]. The severity of DLB correlates with the extent of α-syn pathology, but not with the beta-amyloid burden. Accordingly, some DLB brains exhibit mostly α-syn pathology [20].

## 2. Genetics of Lewy Body Disorders

Similar to other neurodegenerative diseases, LBD are complex multifactorial disorders characterized by an important heterogeneity. Mutations in a single gene cause only a small percentage of cases, and the vast majority develops as a result of the interaction of multiple genetic and environmental factors.

### 2.1. Parkinson’s Disease

Shortly after the discovery of α-syn as the main component of PD-associated pathology, the first mutation in the synuclein alpha gene (SNCA) was identified [21]. Since then, intense studies have unveiled that numerous genes are involved in the development of PD. Although most of these are common genetic susceptibility factors that increase the risk of an individual to develop PD, 5–10% of all PD patients present monogenic forms of the disease [22].

#### 2.1.1. Disease-Causing Rare Variants

Since 1997, more than 20 genes have been reported to cause PD. Of these, SNCA, the leucine rich repeat kinase 2 (LRRK2), VPS35 retromer complex component (VPS35), GTP cyclohydrolase 1 (GCH1) and ataxin 2 gene (ATXN2) show an autosomal dominant inheritance and their role as causative genes has been corroborated by numerous studies [23]. Disease-causing variants in these genes have been found in various families, and the effect of the amino acid changes on protein structure and function has been experimentally confirmed [22,24].

So far, eight missense variants have been identified in the SNCA mutations cluster in exons 2 and 3, and SNCA duplications and triplications have been found in some families [25,26]. Of the more than 100 missense and nonsense variants identified in LRRK2, only nine are considered to be pathogenic [22]. Among them, the most frequent variant is G2019S and accounts for between 1% and 5% of all European PD cases and up to 33% in Northern Africa [27]. In VPS35, a single missense variant, D620N, has been described to segregate with PD in different populations [28]. Although the GCH1 gene is mainly related to dopa-responsive dystonia, some missense variants in GCH1 have been also related to familial PD cases with an early onset of between 40 and 45 years [29]. Moreover, ATXN2 is not only involved in PD development. Whereas the CAG-expansion in ATXN2 causes spinocerebellar ataxia 2, an interrupted expansion of the polyglutamine stretch is responsible for the development of PD [30].

Missense variants in additional genes have been more recently described as PD causing. They include the DnaJ heat shock protein family (Hsp40) member C13 (DNAJC13), transmembrane protein 230 (TMEM230), coiled-coil-helix-coiled-coil-helix domain containing 2 (CHCHD2), RIC3 acetylcholine receptor chaperone (RIC3), GRB10 interacting GYF protein 2 (GIGYF2), HtrA serine peptidase 2 (HTRA2), eukaryotic translation initiation factor 4 gamma 1 (EIF4G1), and ubiquitin C-terminal hydrolase L1 gene (UCHL1) [22,31]. Of these, the most corroborated variant as disease-causing is p.T61I in CHCHD2, since it segregates with the disease phenotype in various families [31,32]. Finally, a recent international study identified rare variants in the LDL receptor-related protein 10 gene (LRP10) in seven families with PD [33]. These findings could be confirmed in an independent study, where four PD patients carried three different variants [34]. Corresponding functional studies additionally suggested that these variants might be causing PD in the affected families [33].

In addition to genes causing autosomal dominant forms of PD, other genes, including the parkin RBR E3 ubiquitin protein ligase (PRKN, PARK2), parkinsonism associated deglycase (DJ-1, PARK7), PTEN induced kinase 1 (PINK1), and F-box protein 7 gene (FBXO7), are responsible for juvenile-onset autosomal recessive PD forms [28,32]. PRKN was identified shortly after α-syn discovery and causes the majority of PD cases diagnosed at a young age [35]. In contrast to PD patients with PRKN mutations, patients carrying FBXO7 variants develop a more aggressive form of the disease [36]. Recently identified loci possibly also causing recessive PD forms are hemizygous deletions of the chromosome 22 region q11.2 [37], and rare variants in the synaptojanin 1 (SYNJ1) [38,39], DnaJ heat shock protein family (Hsp40) member C6 (DNAJC6) [40], podocalyxin like (PODXL) [41], and peptidyl-tRNA hydrolase domain containing 1 gene (PTRHD1) [42]. Finally, an X-linked transmission has been described for the RAB39B, member RAS oncogene family gene (RAB39B) [43], but further studies are needed to confirm its implication in causing PD.

#### 2.1.2. Common Variants Associated with Disease

In addition to the rare variants in disease-causing genes, the association of numerous common variants that confer risk to PD development has been reported. Most of these variants are located in noncoding regions of the genome, and only for a few, their functional role has been investigated [44]. The most studied common variants are located in disease-causing genes, and not only single-gene association studies, but also numerous genome-wide association studies (GWAS) have repeatedly validated that common variants in SNCA and LRRK2 are associated with PD [45,46]. Two other genes persistently found in independent GWAS are GBA (glucocerebrosidase) and MAPT (tau) [46]. Especially, the haplotype H1 of the latter is associated with increased PD risk through expression changes and modification in alternative splicing of MAPT [46,47].

However, of the more than 800 GWAS carried out for PD, only a few yielded consistent results [48].

A recent GWAS meta-analysis revealed the association of 12 loci, including SNCA, LRRK2, GCH1, transmembrane protein 175 (TMEM175), serine/threonine kinase 39 (STK39), transmembrane protein 229B (TMEM229B), branched-chain keto acid dehydrogenase kinase (BCKDK), microRNA 4697 (MIR4697), inositol polyphosphate-5-phosphatase F (INPP5F), Ras-like without CAAX 2 (RIT2), signal-induced proliferation associated 1 like 2 (SIPA1L2), and transmembrane serine protease 9 gene (TMPRSS9) with risk modification for developing PD [49]. These findings, together with numerous on-going studies, are important in order to untangle the genetic architecture of the different PD forms.

### 2.2. Dementia with Lewy Bodies

DLB has been described and recognized only 20 years ago as an independent entity. Its neuropathological overlap with AD leads to a corresponding clinical overlap, and accordingly, it is still challenging to diagnose DLB correctly, leading to elevated under- and misdiagnosis rates. A direct consequence of these difficulties is the lack of large cohorts with certain DLB diagnosis hindering the realization of large genetic association studies [50]. Therefore, much less is known about the genetics of the disease.

However, constant efforts have allowed identification of some rare disease-causing variants in families with members affected by DLB, but also in DLB cases without familial history. Additionally, common variants have been found to modify the risk of developing DLB.

#### 2.2.1. Rare Variants in DLB-Causing Genes

SNCA was the first gene identified as DLB-causing gene. Two missense variants, p.E46K and p.A53T, and SNCA triplications were identified in DLB patients from families with other members affected with PD [51]. Later, two mutations, p.V70M and p.P123H, were detected in the beta-synuclein (α -syn) gene (SNCB). Whereas p.V70M was found in a sporadic DLB case, p.P123H was present in familial DLB [52]. α -syn is known by its nonamyloidogenic characteristics, which are abolished by both variants. In the case of p.P123H, this abolishment occurs through changes within the polyproline-II structure leading to the compaction of the C-terminus [53].

In addition to SNCA and SNCB, both directly related to the development of α-syn pathology, rare variants were also identified in genes that had been related before to AD. These include two variants in presenilin 1 (PSEN1), three in presenilin 2 (PSEN2), one in the amyloid-beta precursor protein (APP), as well as the duplication of APP [54].

Finally, rare variants in the LPR10 gene have been found in DLB patients of large families with members affected with either PD or DLB [33].

#### 2.2.2. Common DLB Risk-Modifying Variants

Similar to rare disease-causing variants, common variants that may modify the risk of an individual to develop DLB or the disease course are located in genes that have been either associated with PD or AD. The genes also described as PD risk modifiers will be discussed below.

The allele ε4 of the apolipoprotein E gene (APOE) is a well-recognized risk factor for AD and has also been studied in DLB cohorts. As in AD, APOEε4 is accumulated in DLB and accelerates the disease course leading to shorter survival of DLB patients [55,56]. Although APOEε4 is overrepresented in DLB, independently on the co-occurrence of AD pathology, the mechanisms through which APOEε4 contributes to the development of dementia seem not to be directly related to beta-amyloid or tau pathology [56]. The APOEε2 allele, on the contrary, shows protective effects against the development of DLB [57]. The K-variant of the butyrylcholinesterase gene (BChE) confers decreased risk for DLB development [58].

### 2.3. Genes Modifying Risk for Both PD and DLB

Since PD and DLB share the development of α-syn related pathology as common disease-related substrate, some genes were identified as genetic risk factors for both.

The most studied of these genes is *SNCA*, and interestingly two distinct association profiles within its locus have been identified: one for parkinsonism and the other for dementia [59,60]. Accordingly, common variants located in the 3’ SNCA portion are associated with PD [61], and variants located in the 5’ SNCA part with DLB. Additionally, a haplotype tagging to two the centrally located SNCA variants is associated with PDD, demonstrating that SNCA variability contributes differentially to PD and DLB [62,63].

β-syn belongs to the synuclein family and has been reported as a natural negative regulator of α-syn aggregation [64]. Correspondingly, the SNCB gene has also been studied as a potential risk factor for synucleinopathies, and common SNCB variants have been identified to modify PD risk [65,66]. Moreover, an SNCB haplotype has been associated with DLB exhibiting concomitant AD pathology, and insertion/deletion variants in the 5’ portion of SNCB confer risk for developing DLB without AD pathology [62].

The scavenger receptor class B member 2 (SCARB2) locus has also been identified as risk modifier, first for PD [67], and later, as a result of the first DLB GWAS, also for DLB [60]. Similar to the SNCA locus, PD and DLB show differential association profiles with the SCARB2 locus [54,59].

Another disease risk modifying locus, common for both PD and DLB is GBA, and only recently, heterozygous GBA variants have been associated with LBD. Whereas an odds ratio of 5.43 has been reported for the association of GBA with PD [68], an odds ratio of 8.28 was found for its association with DLB [69]. Moreover, GBA variants are responsible for an earlier onset of LBD [70], and cause an accelerated disease course, leading to earlier death [71].

## 3. Epigenetics in Lewy Body Diseases

Epigenetics is the denomination of the mechanisms that regulate gene expression and are independent of the primary DNA sequence. Initially, in 1990, Holliday defined epigenetics as the “temporal and spatial control of gene activity during the development of complex organisms” [72]. Now, it is assumed that epigenetic modifications are heritable, but are not based within the DNA sequence per se.

Over the last few years, the understanding of epigenetics is further changing, and it has been proposed that the term epigenetics should refer to changes at the chromosomal level [73,74,75]. Accordingly, Heesbeen and Smidt have suggested that miRNA-regulated gene expression should not be defined as epigenetics [76]. Taking into account these considerations, in the present review, we will summarize and discuss recent findings in regard to changes in DNA methylation and histone modification in LBD. Further, we revised literature on the suitability of epigenetic changes as disease biomarkers, and on the advances of using epigenetic changes as therapeutic targets.

One of the two key mechanisms regulating gene expression is promoter methylation. Methylation occurs at cytosines located 5’ to guanine residues (CpG), that are present at high density within so-called CpG-islands. DNA methylation is mediated by methyltransferases (DNMT) and, whereas DNMT1 is responsible for maintaining DNA methylation during replication, DNMT3a and DNMT3b mediate de novo DNA methylation [77]. When located in promoter regions, hypermethylated CpG islands repress gene expression, whereas hypomethylated CpG islands lead to increased expression. These mechanisms play an active role during development and aging [78,79]. Over the past years, altered methylation patterns have been associated with disease, especially cancer [80], but recently also with neurodegenerative diseases.

The second major epigenetic mechanism involved in gene regulation consists of histone modifications. Histones represent the proteic part of chromatin and allow the compaction of DNA. The main histones are H1–H4, and each nucleosome representing the smallest chromatin unit contains an octamer comprising two of each H2A, H2B, H3, and H4 which the DNA is wound around [81]. H1 is associated with a fragment of linker DNA between two nucleosomes.

Post-transcriptional modification of histones includes acetylation and methylation of lysine residues of H3 and gene transcription is activated with acetylation of H3 lysine residues 4 (H3K4), 36 (H3K36), and 79 (H3K79), and repressed with acetylation of H4 lysine residues 9 (H3K9) and 27 (H3K27), and H4 residue 40 (H4K20) [82]. The balance between two classes of enzymes, histone acetyltransferases (HATs) and histone deacetylases (HDACs), is critical to the correct functioning of chromatin [83]. HDACs comprise four classes, of which class III are sirtuins (SIRT1-SIRT7). Although many functions characterize sirtuins, they have been involved in the etiopathogenesis of neurodegenerative disorders by their function as histone deacetylases [84].

### 3.1. The Alpha-Synuclein Gene SNCA and Epigenetic Modifications

**Figure 1:**
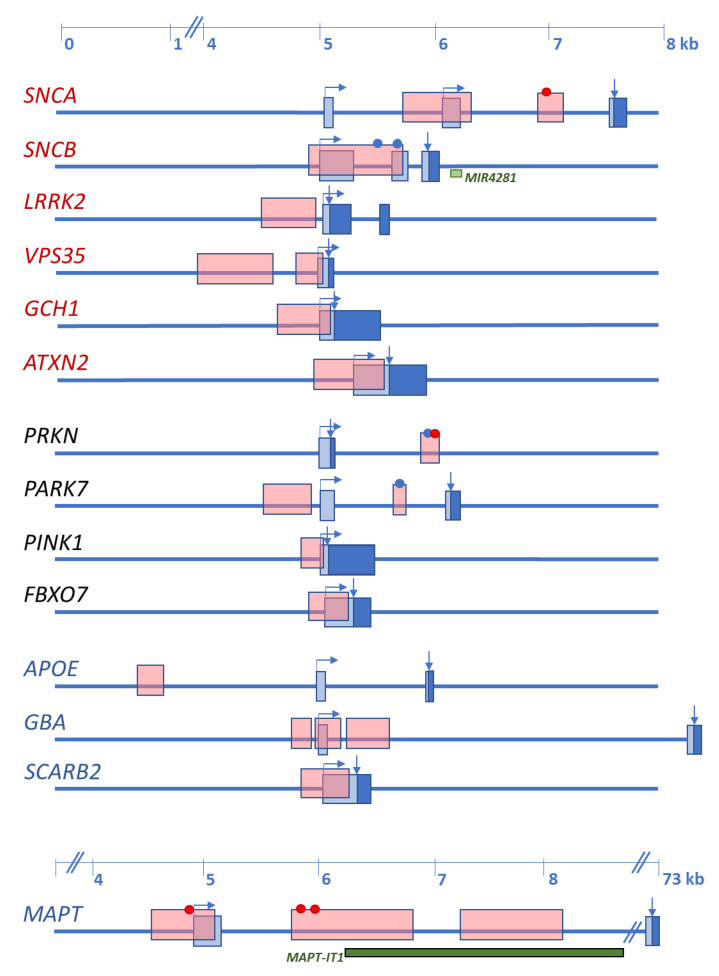
Schematic representation of the initial exon(s) of Lewy body disorders (LBD)-causing and the four main LBD-risk modifying genes with CpG islands. Names of the genes are indicated at the left. In red are autosomal dominant disease-causing genes, in black are autosomal recessive disease-causing genes, in blue are main risk factors, identified by genome-wide association studies (GWAS). Boxes represent exons: light blue—noncoding sequence; dark blue—coding sequence. Horizontal arrows indicate transcription start and direction; vertical arrows indicate the CDS start. Dots indicate CpG islands studied for methylation changes, in red is change of methylation levels, in blue is unchanged methylation. Light green box, miRNA gene within the SNCB sequence. Dark green box, lncRNA within the MAPT sequence.

Since α-syn oligomerization and aggregation are now accepted as the primary pathological events observed at synapses and preceding inclusion body formation, its gene has been extensively studied. Besides mutations within the exon 2/exon3 cluster, numerous common variants and gene multiplication, two CpG islands have been identified within the 5’ portion of the gene. One is localized in the SNCA promoter spanning the sequence preceding and including exon 1, and the other is located in intron 1 (Figure 1).

#### 3.1.1. SNCA Promoter Methylation Changes in PD

Most of the studies have been carried out in cohorts of PD patients, and a persistent hypomethylation mainly of the intron1 CpG island has been reported. Accordingly, the SNCA intron 1 region showed diminished methylation in the SN and the putamen of PD patients when compared to controls, and the majority of specifically hypomethylated CpG sites were located within promoter binding sequences. Correspondingly, the hypomethylation state correlated with increased SNCA expression in PD brain [85]. Pronounced hypomethylation has been found in the SN, but neither in the anterior cingulate gyrus nor the putamen of PD patients, especially of those patients who were suffering from the disease for 20 or more years [86]. An additional study reported that methylation changes of the SNCA intron 1 region in the SN did not correlate with PD, but only eight PD cases and eight controls were included in the study [87]. The intron 1-CpG island of SNCA is also hypomethylated in peripheral blood of PD patients [88,89,90,91]. The most important reduction of CpG methylation was found at specific cytosine residues, especially in PD patients with disease onset before the age of 50 years, but did not correlate with disease stage [88,91]. Additionally, SNCA hypomethylation significantly correlated with a positive familial history of PD [91].

In an early study, SNCA promoter and intron 1 methylation were analyzed in four brain regions and the cerebellum of LBD patients without correlating with the clinical diagnosis of the patients, but with the stage of Lewy pathology [92]. Whereas no differences of overall methylation levels were found between patients and controls, increased methylation was found in the putamen of limbic predominant LBD. Additionally, methylation levels were lowest in the cerebellum compared to the other brain regions. Finally, increased SNCA methylation also correlated with increasing age of the patients [92].

Association analysis between SNCA methylation levels and genetic variation at the SCNA locus revealed that shorter alleles of the complex microsatellite rep1 (D4S3481) are associated with higher methylation levels [89]. Moreover, intron 1 CpG island methylation was decreased in carriers of the G-allele of the SNP rs3756063, in both peripheral blood and brain [90]. Although the increase of SNCA mRNA levels could be expected, only in one of the four studies was such an expression change observed [88,89,90,91]. These inconsistencies could be due to the complex splicing pattern of the SNCA gene, for which more than 16 alternative transcripts have been described (revised in [93]). Different initial exons characterize at least four groups of SNCA transcripts. Accordingly, the extend of intron 1 CpG island methylation could affect their expression differentially. To obtain reliable data regarding the effect of SNCA methylation changes on mRNA expression, the expression of the different transcripts should be analyzed to determine the effect of methylation changes on each one of them.

The exposure to environmental toxins, including pesticides and heavy metals, has been suggested to increase the risk of developing PD. Therefore, various studies explored the effect of such agents on SNCA methylation. For example, 1-methyl-4-phenylpyridinium (MPP+) induced the reduction of DNMT3a and DNMT3b in SH-SY5Y cells, and at the same time, demethylation of the SNCA promoter and overexpression of SNCA mRNA was observed [94]. In a rat model, the exposure to methamphetamine has been associated with the hypomethylation of the SNCA promoter and correlated with α-syn overexpression in the SN [95]. These results were confirmed recently in mice, where the exposure to methamphetamine induced striatal α-syn-related neuropathologic changes [96]. These changes were accompanied by SNCA promoter demethylation and with the increase of α-syn levels in striatal neurons and in limbic areas.

#### 3.1.2. SNCA Promoter Methylation Changes in DLB

Much fewer studies have addressed the SNCA CpG island methylation status in DLB. Recently, Funahashi and colleagues analyzed SNCA intron 1 methylation levels in leucocytes and detected overall diminished methylation in DLB patients compared to controls. Although, this hypomethylation did not correlate with expression changes of total SNCA transcripts giving rise to α-syn 140, SNCA126, an α-syn isoform lacking exon 5, was significantly increased in DLB [97].

#### 3.1.3. The Role of α-syn in Methylation

In addition to SNCA CpG island hypomethylation, α-syn per se can modify the DNA methylation machinery. In α-syn-overexpressing transgenic mice, decreased nuclear levels of DNA methyltransferase 1 (Dnmt1), and its translocation to the cytoplasm has been detected [98]. This finding could be confirmed in human LBD brain, indicating that α-syn, if present in excess, sequesters DNMT1 from the nucleus. Correspondingly, the global diminution of DNA methylation, including hypomethylation of the SNCA CpG islands, has been found in these brains [98]. Accordingly, two docking sites required for the putative interaction with DNMT1 have been recently identified in the CpG island of SNCA intron 1 [99].

#### 3.1.4. SNCA, α-syn, and Histone Modification

Numerous studies have investigated the functional role of α-syn in both physiological conditions and disease. Due to its structural characteristics, α-syn is a multifunctional protein [100], and among the various functions, its involvement in histone modification through methylation- and acetylation changes has been described.

An early study was carried out in a transgenic Drosophila PD model, where overexpressed α-syn colocalized with H3 on polytene chromosomes [101]. Thus, α-syn masks H3 acetylation sites by direct interaction and additional interaction with the deacetylase SIRT2, promoting H3 hypoacetylation. Moreover, SNCA expression was analyzed in a patient carrying the SNCA p.A53T variant [102]. Expression studies were performed in a cell line and blood of the patient and revealed the silencing of the p.A53T allele. At the same time, the expression of the one remaining normal SNCA allele was higher than the expression of the two normal SNCA alleles in control subjects. The silenced p.A53T allele could be reactivated by the treatment of cells with histone deacetylase inhibitors, showing an association between mutant α-syn and histone modification [102]. 

The effect of α-syn on histone modifications was further investigated using transgenic Drosophila and inducible SH-SY5Y neuroblastoma cells [103]. The overexpression of α-syn in these models led to increased mono- and dimethylation of H3 specifically involving H3KThis hypermethylation was preceded by the increase of lysine N-methyltransferase 2 (EHMT2) mRNA, and the subsequent mRNA decrease of REST complex members was found. The latter observation confirmed that α-syn overexpression modifies H3 directly through H3K9 methylation [103]. Additionally, an H3K27 acetylation-enriched enhancer sequence was identified at the SNCA locus [104].

Finally, the overexpression of α-syn in dopaminergic neuronal cells leads to an important deregulation of gene expression in these cells. An elevated percentage of the downregulated genes were genes involved in DNA repair [105]. Therefore, the possible association with histone modification was investigated, and decreased H3 acetylation was found in α-syn-expressing cells [105]. Altogether these studies underline the complex involvement of α-syn as a trigger of LBD development.

### 3.2. Gene-Specific Promoter Methylation

As discussed in Section 2, five genes have been repeatedly shown to cause autosomal dominant forms of PD and four to cause juvenile-onset autosomal recessive PD. In contrast, only two disease-causing genes have been identified for DLB. Additionally, *GBA*, *MAPT*, and *SCARB2* variants act as disease modifiers for both PD and DLB, and *APOE*ε4 is a risk factor for DLB. Figure 1 shows that all these genes contain CpG islands in their promoter regions or the region preceding the transcription start.

However, only a few of these CG regions have been studied in regard to their methylation status and possible changes related to LBD.

#### 3.2.1. Promoter Methylation Change of Disease-Causing Genes

Apart from SNCA, which is the most studied gene in LBD, possible promoter methylation changes have been analyzed only for ATXN2, PARK7, and PRKN.

The ATXN2 CpG island has been studied in the context of spinocerebellar ataxias (SCAs) and differences in the disease course of SCA2 patients correlated with the different methylation level of the ATXN2 promoter [106]. The lower the methylation levels, the earlier the disease started to develop in the affected individuals. Although this region has not been examined in the context of PD, the results of the study indicate that ATXN2 promoter hypomethylation may play an important role in modifying both the onset and course of PD.

PARK7 contains two CpG islands. Similar to SNCA, the first spans exon 1 (CpG1) and the second is located in intron 1 (CpG2) preceding the transcription start (Figure 1). So far, no differential promoter methylation levels have been found in PD, but only one study has been carried out in peripheral blood [107].

PRKN promoter methylation has been analyzed in three independent studies. In one, three brain areas, including the occipital cortex, SN, and cerebellum, from five PD patients and two controls were examined, and methylation of only one individual CpG was detected in one of the PD cases [108]. Similarly, in the second study carried out in peripheral blood of early-onset PD with and without PRKN mutations, overall hypomethylation was found in both PD patients and controls [109]. In contrast, in the third study, also performed in peripheral blood, hypomethylation of the PRKN promoter was observed in PD patients [91]. However, different methylation detection methods were used in the three studies, which could be the cause of these contradictory results.

Finally, we have analyzed the methylation status of the SNCB gene in postmortem frontal and temporal cortex samples of DLB brains and did not identify methylated CpG-sites within the SNCB promoter CpG island [110].

#### 3.2.2. Promoter Methylation Change of Risk-Modifying Genes

The MAPT promoter-exon1-intron1 region contains four CpG islands located close to each other (Figure 1). Methylation levels in this region have been analyzed in brain and blood of PD patients and were compared to controls [111,112]. In brain, hypomethylation was detected in the putamen of PD patients. No differences in the methylation level were detected in the anterior cingulate gyrus, but hypermethylation was found in the cerebellum of PD patients compared to controls. In blood, MAPT promoter methylation levels correlated with disease onset, the younger the patient at PD onset, the less methylated cytosines were detected [111].

The APOE gene contains a CpG island approximately 4000 bp downstream to exon 1 (Figure 1), which has not been studied so far in the context of LBD [113]. Additionally, the presence of a second CpG island in exon 4 has also been reported [114]. Exon 4 contains the two SNPs (rs7412, rs429358) that define the common APOE genotype comprising alleles ε2, ε3, and ε4, and the presence of the ε4 allele corresponds to a higher CpG density in this region [115]. Although this CpG island presents hypomethylation in the frontal cortex of DLB and AD brains [113], APOE mRNA expression does not correlate with the methylation status of the CpG island of exon 4 [115].

Additional studies addressing methylation changes in LBD risk-modifying genes have also been carried out. For example, TNF promoter methylation was analyzed in cortex and SN samples of PD patients and controls. Although no differences between PD and controls were detected, significantly lower methylation levels were found in the SN compared to the cerebral cortex [116]. In another study, the methylation status of the dopamine receptor D2 (DRD2) promoter was analyzed in blood of DLB and PD patients and compared to controls, and differential methylation changes were detected for both. Whereas CpG1, CpG2, and CpG6 of the island were hypermethylated in DLB, CpG4 was hypomethylated in PD [117].

### 3.3. Histone Modifications

The development and progression of neurodegenerative diseases have been associated with a shift in HAT/HDAC activity, leading mainly to histone deacetylation [83]. Histone remodeling has also been observed in PD, and the ability of HDAC inhibitors on restoring histone acetylation levels highlights the importance of HDAC deregulation as a pathogenic mechanism in LBD [118].

#### 3.3.1. Histone Remodeling in Early LBD

So far, histone remodeling and modification have not been studied in depth in the context of Lewy body diseases. On one hand, no studies have addressed this question in DLB, and on the other, only a few studies have been carried out for PD (see Section 4.4). Although histone deacetylation is accepted to be involved in the pathogenesis of LBD, neither brain area, nor disease stage-specific studies have addressed the role of histone remodeling during the disease course.

#### 3.3.2. Histone Modification Related to Disease-Causing and Risk-Modifying Genes

Besides α-syn, which binds histones masking their acetylation sites, LRRK2 is also directly involved in histone remodeling [119]. After direct binding of LRRK2 to HDAC3, the latter is phosphorylated at Ser-424, increasing its activity. Furthermore, LRRK2 promotes the translocation of phosphorylated HDAC3 to the nucleus, leading to the deacetylation of H4K5 and H4K12, and the corresponding repression of gene transcription [119].

HDAC3 phosphorylation at Ser-424 specifically in neurons is mediated by Pink1 and increases HDAC3 activity in these cells. Phosphorylated HDAC3 interacts with p53, mediates p53 hypoacetylation inhibiting its expression and at the same time, p53-mediated dopaminergic cell death [120].

Some studies demonstrate that histone modifications regulate specific PD genes and related molecules. Besides SNCA (see Section 3.1), MAPT is another PD-associated gene regulated by histone modifications, namely by the methylation of specific lysine residues. The MAPT haplotype H1 is preferentially associated with histone H3K4 trimethylation (H3K4me3), whereas the H2 haplotype is associated with the repressive histone H3K27 trimethylation (H3K27me3) [121]. On the other hand, HDAC4 shows a significant increase in methylation with PD progression. However, it is unknown if these changes are due to prolonged levodopa treatment [122].

Whether other LBD related proteins are also involved in histone remodeling remains unknown.

## 4. Epigenetic Pattern as LBD Biomarker

### 4.1. Importance of Identifying Biomarkers for LBD

Currently, the definitive diagnosis of LBD is achieved postmortem by the analysis of brain tissue. However, at this stage, the information about alterations that occur during disease progression is no longer available. Therefore, recent research has focused on finding more easily accessible tissue, such as blood, plasma, serum, cerebrospinal fluid (CSF), or saliva, that may reflect the changes that are produced in the brain during earlier disease stages or even before symptoms become evident.

Biomarkers are molecules that represent or indicate the particular signature of a physiological or pathophysiological state and that can be easily accessed and quantified [123]. During the last decade, the importance of identifying biomarkers for LBD has acquired a tremendous relevance, first to monitor disease progression and treatment outcome, and second to identify individuals at prodromal disease stage [124]. So far, dopamine imaging is achieved by DaTSCAN, an expensive and invasive radio imaging technique that permits the identification of LBD with high specificity and sensitivity [125].

Since the epigenome is partially dynamic, recent investigations suggest that epigenetic marks may be a new source of biomarkers for LBD [126]. The research performed to date has focused on the identification of altered DNA methylation of LBD related genes involved in candidate pathways to cause LB pathology including oxidative stress, neuroinflammation, lysosomal dysfunction, and cell loss in localized brain regions.

### 4.2. Limitations in the Identification of Epigenetic Patterns as Biomarkers

Obtaining useful biomarkers is challenging since an ideal biomarker should be reproducible in different laboratories, within different cohorts, and reflect brain-related changes in a more easily accessible tissue. The experimental design is crucial from the beginning to minimize bias from factors related to sample selection and processing, the biomarker detection method, and to confounding experimental variables regarding biological and nonbiological batch effects.

Probably, the most important challenge in epigenetic analysis is to achieve a homogeneous DNA source. Different projects (NIH Epigenomics Roadmap and ENCODE) demonstrated that epigenetic patterns are significantly different between tissues, tissue subregions, and cell types within an organism [127,128], and can also be susceptible to circadian fluctuations [129]. For example, de Boni and colleagues observed variation in the methylation levels between different PD brain areas, with the most methylated being the cortex, and the cerebellum being lowest, [92]. Matsumoto and collaborators found hypomethylation in the SN, but not in the anterior cingulated cortex or putamen [86]. Young and colleagues, however, detected a predominance of methylation changes in the dorsal motor nucleus of the vague in comparison to the cingulate gyrus and SN [130]. This differential methylation status has also been described for specific genes in PD samples, i.e., MAPT is hypomethylated in the putamen, but hypermethylated in the cerebellum [111]. In addition, Li and colleagues demonstrated hemispheric brain asymmetry at epigenetic, transcriptomic, and proteomic levels associated with the lateralization of PD symptoms, exhibiting the symptom-dominant side as having increased methylation [131].

**Figure 2:**
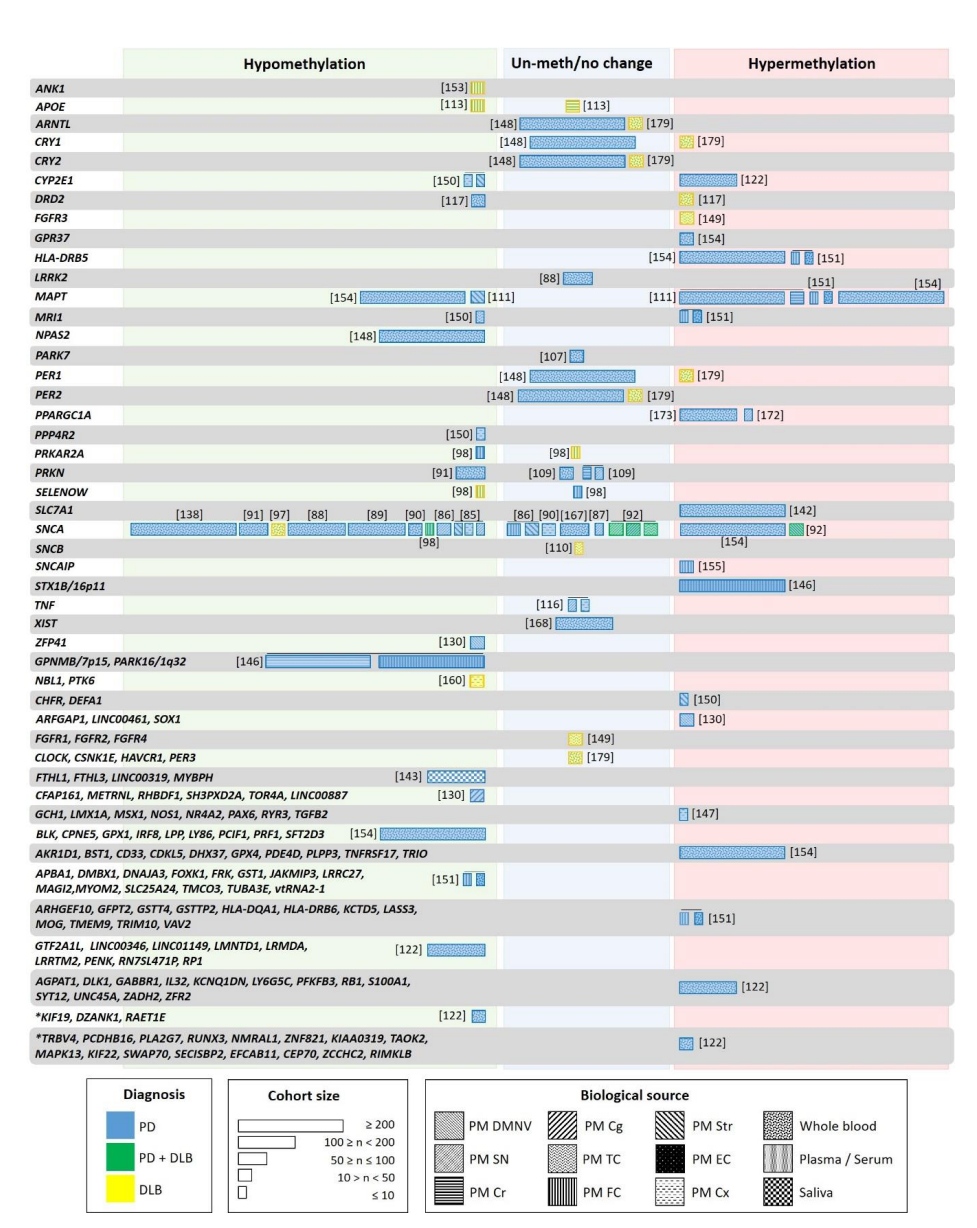
Genes studied for differential methylation in patients with LBD. Each box indicates one individual study, with width proportional to the sample size of each study. The color of the boxes indicates the patient type: blue—PD; yellow—DLB; green—PD and DLB. Box patterning shows the biological source used in each study: Postmortem dorsal motor nucleus of the vagus (PM DMNV), substantia nigra (SN), cerebellum (Cr), temporal cortex (TC), frontal cortex (FC), Striatum (Str), entorhinal cortex (EC), cortex (Cx), whole blood, plasma/serum, peripheral blood and saliva. Asterix indicates genes with longitudinal changes in methylation in patients not receiving L-dopa/entacapone.

Aging, genetic, and environmental factors amplify this divergent epigenetic pattern, which is dynamic over time. On one hand, epigenetic variation accumulates in aging cells, affecting genomic locations differentially. Whereas promoter associated CpG islands undergo an age-related methylation increase, areas with highly methylated DNA methylation, such as repetitive elements in intergenic regions, tend to lose methylation. These DNA modifications take place rapidly in early life, and gradually slow down over the life span (reviewed in [126,132]). Moreover, this epigenetic aging is believed to be accelerated in neurodegenerative diseases [133]. On the other hand, epigenetic pattern changes are associated with the disease state. This has been observed in the first longitudinal methylation analysis performed in PD patients, where DNA methylation dynamics were associated with disease progression, and methylation rate changes ranged between 1.5% reduction and 1.7% increase per year [122]. The genes exhibiting longitudinal methylation changes in PD are shown in Figure 2. These data suggest that the adequate characterization of LBD patients considering the associated Braak or McKeith stages is mandatory. Independently, Li and colleagues showed that the epigenetic asymmetry observed between the brain hemispheres was reduced with aging in PD, indicating its contribution to bilateral symptomatic progression in PD [131]. Besides aging, epigenomic alterations can be produced by genetic factors as well. There are numerous sites in the genome that exhibit allele-specific epigenetic differences, which are haplotype dependent, highly tissue-specific, and prevalent in the brain [134,135].

Despite the difficulty for the acquisition of well-characterized and well-balanced samples, the analysis of large cohorts minimizes this intraindividual variability. Moreover, since factors like the exposure to pesticides and endocrine disruptors (i.e., paraquat), diet (such as folate, coffee) or physical exercise seem to modify DNA methylation, these should be considered in the experimental design [132,136,137]. Since prolonged levodopa treatment affects the methylation levels [138,139] and H4 deacetylation [122,140], the medication history should also be considered. Confounding factors can also be the origin/geographic distribution of patients [141] or gender. For example, gender-specific methylation pattern has been described for MAPT [111].

Another alteration confounding the relationship between the disease and epigenetics is blood cell composition, which differs significantly between PD cases. Correspondingly, many of the genome wide significant CpGs correlate with changes in cell composition [122,133,142,143]. Cell composition in specific brain areas can differ between patients and controls due to degeneration and cell loss characteristic in neurodegenerative diseases [130].

A biased result can also be obtained by the use of an unappropriated method for the detection and analysis of epigenetic marks. When choosing a method, several key factors should be considered, such as the aim of the study, sample quality and manipulation, or the requirements of sensitivity and specificity of the study. In the studies performed to analyze DNA methylation, different approaches have been used to identify and quantify changes, all of them with advantages, but also with limitations (i.e., different sensitivity, percentage of coverage), which can be a source of variability (reviewed in [144]).

### 4.3. DNA Methylation Pattern as a Potential Biomarker for LBD

DNA modifications represent a highly promising biomarker for neurodegenerative disorders [145]. As discussed above, DNA methylation in LBD has been primarily investigated within selected candidate genes [85,86,113,116,117,146,147,148]. However, global DNA methylation abnormalities in PD and DLB brains have been identified in several epigenome-wide studies (see Figure 2) [122,130,131,142,149,150,151,152,153,154,155].

Neurodegenerative diseases, including AD, DLB, PD, and Alzheimer-like neurodegenerative profile associated with Down’s syndrome, share common epigenomic patterns, with similar aberrant CpG methylation in common promoters. These observations suggest that these diseases might share similar initial pathogenetic mechanisms that subsequently evolve into different clinical entities with different molecular and cellular features [153]. Accordingly, a common promoter methylation pattern was found for these four neurodegenerative diseases involving in the Erb, TGF-beta, Hippo, Wnt, MAPK signalling pathways, among others. Additionally, PD and DLB shared promoters with altered methylation of the phosphatidyl inositol, PI3K-Akt, and mTOR signaling pathways. These findings are extensively described and documented in the review provided by Delgado-Morales and Esteller [156]. Additionally, an independent study identified a global hypomethylation state in postmortem DLB and PD brain samples [98]. In another independent cohort of postmortem brain samples, 1428 differentially methylated regions were common in PD and DLB [153]. Epigenetic investigation in pluripotent stem cells (iPSC)-derived dopaminergic neurons (DAn) from PD patients showed a commonly shared global DNA hyper-methylation in monogenic as well as sporadic PD cases [157,158] re-enforcing the idea of a common aberrant DNA methylation in LBD.

The global hypomethylation observed in LBD was attributed to the translocation of DNMT1 from the nucleus to the cytoplasm [98]. This hypomethylation located at specific promoters [90,111,113,117,148,150,151] could seem contradictory to the global PD hypermethylation reported in iPSC-derived dopaminergic neurons (DAn) from PD patients [157,158]. However, whereas hypomethylation was found mainly in promoter and gene regions, hypermethylation corresponded mainly to intergenic noncoding regions. Recent studies suggest that the latter could play a role in pathogenic processes of human disease by affecting regions involved in transcription regulatory or noncoding transcripts [159]. Fernández-Santiago and colleagues reported a deficit in a transcription factor network in PD DAn, relevant to the pathology (FOXA1, NR3C1, HNF4A, FOSL2). This deficiency could mediate genomic hypermethylation in specific regions as a result of a functional imbalance in the enzymatic machinery regulating DNA methylation [157]. Other studies reported methylation changes in RNA genes such as long intergenic non-protein coding (LINC) and miRNAs in PD patients (Figure 2) [122,130,143,151,154]. However, only LINC00461 was found to be hypermethylated [130].

Both, PD and DLB present distinctive DNA methylation patterns that can be differentiated from control subjects, but to date, there is not enough knowledge to allow discerning between them (Figure 2) [122,130,147,151,152,154,160]. In recent genome-wide studies, a large number of differentially methylated regions has been identified. However, there is only a slight overlap with previous reports, and many of the results have not been validated in independent cohorts (See Section 4.5). For instance, the synuclein alpha interacting protein (SNCAIP) gene region was hypermethylated in cortical samples of a small PD cohort [155]. However, these findings were not corroborated independently, and neither SNCAIP expression levels were analyzed in the same samples. Other examples are the neuron-specific methylome analysis carried out in the inferior temporal lobe of LBD brains that showed hypermethylation of the fibroblast growth factor receptor 3 (FGFR3) gene [149]. This hypermethylation correlated with FGFR3 protein overexpression in the same samples and could represent the response to α-syn neurotoxicity. However, this finding has not been replicated. The genome-wide DNA methylation profiling studies that revealed the hypermethylation of the solute carrier family 7-member 11 (SLC7A11) promoter in blood correlated with diminished SLC7A11 expression in a large PD cohort [142]. Only when gene/pathway functional enrichment analysis is performed, are the results revealed that the observed methylation changes may contribute to alterations in neurogenesis, neurodevelopment, neurodegeneration, immunity, and stress oxidation [122,131,147]. Among them, the Wnt signaling pathway, involved in immune function and dopaminergic cell fate and functioning, was the most repeatedly reported [130,147], and which has also been associated with PD at genetic and expression levels [161,162,163]. In DLB, the alteration of several pathways, such as MAPK, ErbB, neurotrophin, mTOR, p53 signaling, and regulation of the actin cytoskeleton, has been reported [153].

The DNA methylation pattern has also been studied in the mitochondrial genome. Loss of methylation in nearly all CpG sites in the noncoding displacement (D) loop region of the mitochondrial DNA (mtDNA) has been observed in the SN in PD cases compared to controls [164]. Coppedé and Stoccoro review in more detail the possible connections between mitoepigenetics and neurodegenerative processes. They conclude that mitoepigenetic changes could contribute to neurodegeneration by the high number of mitochondria found in neurons corresponding to the need for energy production. Therefore, neurons are particularly vulnerable to the accumulation of mtDNA mutations with aging. Moreover, epigenetic changes in mtDNA have been associated with environmental toxins, oxidative stress, drug treatment, disease, and aging [165].

Information on methylation changes in non-neurological tissue of LBD patients is still scarce. Despite concerns regarding the use of whole blood for DNA methylation profiling (due to variability as a consequence of the complex mixture from different cell types and the high variability among individuals), a meta-analysis reported a significant covariation between brain and blood methylomes. In particular, the analysis revealed a robustly defined age-related comethylation module suggesting that blood could be a promising surrogate for the brain when studying the effects of age on DNA methylation profiles [166]. When comparing postmortem frontal cortex with leukocytes from the same individuals (PD patients and control subjects), Masliah and collaborators identified concordant methylation alterations in a subset of genes previously implicated in PD pathology (Figure 2). CpG- hypomethylation was detected for more than 80% of the analyzed genes. Although the size of the studied groups was small (five and six individuals, respectively), a similar methylation pattern between both tissues was detected, re-enforcing the idea that leukocytes might truthfully reflect brain-associated changes and represent an acceptable source for biomarker discovery in PD [151].

However, the identification of biomarkers is difficult since similar conditions must be guaranteed in different studies (see Section 4.2). Many studies have explored the suitability of epigenetic changes as possible biomarkers, but only a few have rendered consistent results (Figure 2). For example, although five independent studies showed reduced SNCA intron 1 methylation in peripheral blood of PD patients [88,89,91,97,138], these results could not be reproduced in another study based on leukocyte DNA [167], and even increased methylation was detected when studying peripheral blood mononuclear cells (PBMC) [154]. A similar discrepancy was also seen in studies of brain tissue [87,92].

The first studies analyzing the methylation level in specific genes or performing epigenetic-wide association studies seemed to indicate similar methylation changes in PD blood and brain. However, recently, 24 differentially methylated regions were identified in a cross-sectional genome-wide methylation analysis performed in PD blood. One of these regions contained 13 hypermethylated CpG sites in the cytochrome P4502E1 (CYP2E1) promoter (Figure 2) [122]. The analysis CYP2E1 expression in brain showed an increase instead of the expected decrease [150].

Studies performed in PBMC comparing epigenome-wide DNA methylation in siblings and monozygotic twins discordant for sporadic PD did not show significant differences in the methylation pattern of more than 90 PD-related genes [154,168]. An extensive heterogeneity was observed among the patients, indicating that methylation changes are associated with phenotypic variability in PD [154]. Only GPR37 was differentially methylated in the affected siblings of monozygotic twins, and 26 genes showed differential methylation when comparing PD patients and controls (Figure 2). Among these, MAPT, PDE4D, GPX1, GPX4, had been identified as risk loci for PD in a GWAS, but only PDE4D has been replicated in an independent cohort [154]. These results indicated that PD risk could arise from the combination of several demethylated genes.

Only one study has addressed methylation changes in saliva of PD patients and showed that differential methylation patterns differ between blood and saliva. However, both are associated with PD, and mainly mitochondria-related genes, and genes with cytoskeleton function composed these patterns (Figure 2) [143]. Whereas genes involved in neuron differentiation and the Wnt receptor signaling pathway were differentially methylated in blood, in saliva, these were genes related to neuron differentiation and projection. Similar pathways had been detected as affected by differential methylation in the brain. Finally, a significant association between methylation changes in the RNA gene LINC00319 in saliva and PD was found [143].

### 4.4. Histone Modification Patterns as Alternative Epigenetic Biomarkers for LBD

In general, little information is available on brain histone modifications of LBD patients. Various studies reported increased acetylation in PD-related samples. One assessed net acetylation of H3 at H3K9, H3K14, H3K18, and H3K23 in the primary motor cortex of PD patients with early disease, classified as stage 3 and compared to controls. Overall elevated histone H3 acetylation levels were found in PD brains and were due to increased H3K14 and H3K18 acetylation. In contrast, the lysine residue at position 9, H3K9, was hyperacetylated in PD [169]. The second study reported increased histone acetylation (H2AK5, H2BK15, H3K9, and H4K5) and lower levels of HDAC in midbrain dopaminergic neurons isolated from PD patients. This increase, however, was not as relevant in brain tissue or the cerebellar cortex [170]. Finally, increased histone acetylation was also observed in the SN from early and late PD cases compared to controls. The increase was lowest at early disease stages and accumulated with disease progression. Since in vitro studies revealed that degenerating dopaminergic neurons exhibit histone hypoacetylation and activated microglia histone hyperacetylation, the apparent inconsistency of hyperacetylation could be due to the effects of dopaminergic neurodegeneration and microglial infiltration [171].

The results of these studies underline the need for systematic studies to determine the dynamics of histone remodeling in the different brain areas during the development and progression of LBD. Only then will the effective application of histone acetylation-modifying therapies be possible.

### 4.5. Candidate Biomarkers for LBD

An altered epigenetic pattern of any disease-related gene from a sample of PD or DLB patients showing adequate specificity and sensitivity, as well as consistency in different cohorts and laboratory analysis, could represent a useful biomarker. In the context of LBD, such a biomarker should discriminate between the different Lewy body diseases and controls, and reflect the changes occurring in the brain.

As discussed in the previous sections, many studies have attempted to identify epigenetic marks as biomarkers for PD or DLB. However, the results have not always been consistent. In the case of DLB, only a few methylation studies have been performed, and these reported different demethylated genes. These studies should be replicated by independent cohorts but analyzing similar methylation-prone regions. A much larger number of studies has been carried out for PD. Special attention should be paid to the repeated results obtained in different and independent studies. For instance, regarding the genes with aberrant methylation, PPARGC1A (the gene encoding PGC-1α) and HLA-DRB5 (involved in neuroinflammation and immune system) could be suitable PD biomarker candidates. Both have been reported as hypermethylated in independent studies and in different tissues: PPARGC1A in the SN and peripheral blood [172,173], and HLA-DRB5 in peripheral blood and frontal cortex [151,154] (Figure 2). HLA-DRB5 is also hypermethylated in AD [174]; additional studies should determine the sensitivity and specificity of this potential biomarker for PD.

**Figure 3:**
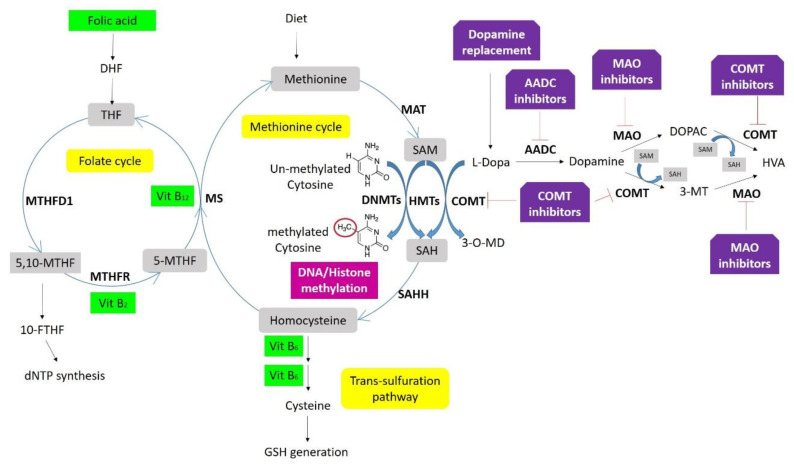
One-carbon metabolism, DNA/histone methylation, and drug interaction. Folate is the center of one-carbon metabolism, which involves three biochemical processes, the folate cycle, methionine cycle, and trans-sulfuration pathway. A set of enzymes (in bold) and coenzymes (in green boxes) catalyzes the different reactions. DNA methyltransferases (DNMTs) control DNA- and histone methylation through methyl groups from SAM. Deficits in folic acid and vitamins B12, B6, and B2 increase homocysteine levels inhibiting DNMTs. Dopamine replacement therapies supply L-dopamine that is converted by either catechol-O-methyltransferase (COMT) into 3-O-Methyldopa (3-OMD) by consuming methyl groups from S-adenosylmethionine (SAM), or aromatic L-amino acid decarboxylase (AADC) to generate dopamine. This dopamine can be metabolized into two substances, 3-ethoxyxtyramine (3-MT) and 3,4-Dihydroxyphenylacetic acid (DOPAC), both converted into homovanillic acid (HVA) and excreted in the urine. Both reactions are catalyzed by COMT using methyl groups from SAM, and by monoamine oxidase (MAO).

Neurodegeneration-associated proteins such as APP and α-syn are related to the methylation markers S-adenosylmethionine (SAM) and S-adenosylhomocysteine (SAH). Accordingly, total homocysteine and the SAM/SAH ratio have been proposed as biomarkers for the DNA methylation potential in PD [175]. Intracellularly, SAM is the major methyl donor for DNA methylation, and SAH the demethylation product of SAM, and at the same time, an inhibitor of DNMT1 activity. Therefore, the magnitude of DNA methylation in a cell is directly associated with the physiological ratio of SAM/SAH and is determined by homocysteine concentration, which depends on the availability of 5-methyl tetrahydrofolate (THF) in the one-carbon metabolism (Figure 3) [132]. Unbalanced SAM/SAH ratio can lead to aberrant methylation reactions, and alterations of this ratio, as well as increased homocysteine, have been found in PD [176]. However, changes in this ratio have also been proposed as a biomarker for atherosclerosis or AD, where lower SAM CSF levels were found in APOEε4 carriers [177,178].

On the other hand, the effects of PD therapy may be associated with expression changes in target genes. Albeit this fact can be inconvenient for studies focused on the identification of demethylated genes associated with LBD, for other studies it is the key for the detection biomarkers that allow the evaluation of both the treatment response and outcome over time. In this sense, Henderson-Smith and collaborators analyzed the longitudinal methylation changes in PD cases with levodopa treatment. Whereas significant time-dependent methylation decrease was found in MATR3, GTF21, ZNF544, LINC00163, BCAN, RP11-300A12, PDGFRB, and PCDH1, it was increased in RIMBP2, Y-RNA, HDAC4, NADK, MTA1, and ZNF623 [122].

Methylation differences in the SNCA promoter have been repeatedly found, and this altered methylation has been proposed as a biomarker for PD. However, similar methylation changes have been observed in both PD and DLB, indicating that these could be a biomarker for synucleinopathies rather than for discerning between them [98]. Although PD and DLB seem to share common epigenomic patterns, they also present differently methylated regions. In particular 13,083 differentially methylated regions have been identified in DLB, and 15,123 in PD postmortem brain tissues. These were specific for each disease and did not overlap with AD, but a list of the genes overlapping with these regions has not been provided, and only a small number of cases was included in the study [153]. A study in human frontal cortex showed PRKAR2A promoter hypomethylation in PD, but not in DLB, and SELENOW hypomethylation in DLB and not in PD (Figure 2) [98]. CRY1 and PER1 present different methylation levels in LBD, being hypermethylated in DLB leukocytes, but unchanged in PD and controls (Figure 2) [148,179].

Additionally, DRD2 DNA methylation rates are increased in DLB leukocytes and decreased in PD. However, DRD2 mRNA expression could not be assessed, because of minimal, nonquantifiable DRD2 expression in leukocytes. Even so, the opposite results of DRD2 methylation should be further explored [117].

The biomarker candidates discussed above should be considered carefully, since all of them have been identified in single studies. Well-designed replication studies are needed to validate the initial data and to identify strong biomarker candidates for PD and DLB.

Contradictory results reported by different studies may be due to the analysis of different tissues, small cohorts, or using different techniques. The comparison of similar patient groups is also required (see Section 4.2). On the other hand, there is a major conceptual problem for the identification of risk factors for neurodegenerative diseases since any of the healthy individuals might develop PD or DLB later in life [176]. In addition, when searching for a biomarker, it should be noticed that postmortem studies represent the endpoint of the disease, and as epigenetic marks may vary according to disease progression, the disease stage should be strictly taken into account in each study [122]. LBD are heterogeneous diseases with variable symptomatology and complication. Therefore, it is impossible that only one biomarker will allow discerning between LBD and their different forms. Possibly, each disease, depending on the stage, age, and other genetic factors of the patients will be characterized by a different mark, underlining the importance of the exhaustive patient characterization. In this sense, several studies proposed a combination of epigenetic marks as a biomarker, and it has been demonstrated that diagnostic accuracy improves when multiple biomarkers are combined in a panel [180]. For example, using a gene expression and methylation data integration analysis approach, Wang and collaborators identified a blood-based 53-gene signature based on hypomethylated regions. The expression of these genes was upregulated so that this signature could represent a biomarker for PD [181]. Independently, a signature of eight CpGs was also identified in blood cells, but with a discriminatory power (AUC) of 0.77, which is too low for clinical use [154]. Finally, the use of seven CpG sites in blood was tested to differentiate DLB and PD, and a sensitivity and specificity of 83.8% and 90.9%, respectively, were obtained [117].

## 5. Epigenetic Changes as a Therapeutic Target for LBD

### 5.1. Current Treatments

Currently, there is no cure for any of the LBD, only treatments able to relieve the symptoms and improve the quality of life are available. There are not even medications approved by the US Food and Drug Administration (FDA) for the treatment of DLB, and drugs approved for other indications, such as AD and PD, are often used [182,183].

Details on all treatments tested or in use for DLB and PD are presented elsewhere [182,183,184]. Combination therapy is applied in most PD cases. Supportive therapies, such as rehabilitation, language therapy, or occupational therapy are combined with drugs including dopamine replacement drugs, dopamine agonists, catechol-O-methyltransferase (COMT) inhibitors, monoamine oxidase B (MAO-B) inhibitors, glutamatergic NMDA receptor antagonists, anticholinergic molecules, and immunomodulatory drugs [185].

Current pharmacological treatment of DLB addresses the improvement of cognitive symptoms, neuropsychiatric symptoms, parkinsonism, sleep disorders, and autonomic dysfunction. Cholinesterase inhibitors are the mainstay of treatments for DLB to improve cognition [182,183]. It is difficult to treat the neuropsychiatric symptoms associated with DLB because of the elevated risk of neuroleptic sensitivity [5,183]. Therefore, nonpharmacological interventions or the use of anticholinergic drugs are recommended. Parkinsonian symptoms usually present in DLB patients are managed similarly to PD, although DLB patients are often less responsive to these drugs. At the same time, these can cause visual hallucinations and other psychotic symptoms, exacerbating existing symptoms [183]. Sleep disorder is treated, combining clonazepam and melatonin, and autonomic dysfunction may improve with standard therapies [186].

### 5.2. Effects of Prolonged Treatments and L-Dopa-Induced Methylation Changes

While treatments to improve the motor symptoms in LBD may be effective, especially at early stages, prolonged use may result in adverse effects [184,185]. For example, dopamine replacement therapies and/or COMT inhibitors directly impact one-carbon metabolism, consuming methyl groups that are required for DNA methylation and increasing homocysteine levels, which directly inhibits the activity of DNA methyltransferases (Figure 3) [122]. In addition, these medications also show in off-target effects, resulting from their delivery to brain areas other than the striatum. These off-target effects are thought to be the basis for the neuropsychiatric adverse effects that can occur, including hallucinations and impulse control disorder [122,184].

Recently it has also been shown that PD therapy per se may alter the DNA methylation state, converting it to one of the factors that may directly contribute to the modification of methylation levels in promoter regions [138,140,154]. Motor symptoms in PD improve with levodopa treatment [187], and since many PD cases have an extended disease course, levodopa therapy becomes chronic. Associated to the prolonged treatment with this PD drug, PD patients may develop levodopa-induced dyskinesia (LID). Recent research in rats with unilaterally lesioned nigrostriatal dopaminergic neurons and posterior treatment with L-dopa revealed that DNA demethylases, but not DNA methyltransferases are upregulated in the striatum. These observations suggest that sustained levodopa treatment may contribute to DNA demethylation in PD and, correspondingly, to changes in gene expression [139]. Methylation changes were found mostly in the regulatory regions of genes which are known to be deregulated in LID [188]. Functional protein cluster analysis revealed that these genes are relevant to mechanisms of synaptic plasticity, uncovering the molecular mechanism of sustained levodopa therapy [139].

### 5.3. Epigenetic Drugs and Their Protective Role

As discussed throughout the review, part of LBD pathophysiology is due to epigenetic modifications. Since epigenetic modifications in neurons are dynamic and reversible, they represent appropriate targets for therapeutic intervention. In fact, the FDA approved the use of epigenetic drugs in cancer after demonstrating that they are able to reverse successfully several epigenetic marks and disease symptoms [189].

Possible targets of these small-molecule epigenetic modulators are components of the epigenetic machinery such as DNMTs, TET family of DNA demethylases, HDACs, and HATs [190]. In this sense, in PD, there are two major epigenetic pharmacological drug-based therapies: those based on DNMT and HDAC inhibitors [191]. DNMT inhibitors such as 5-aza-2’-deoxycytidine (5-aza-dC) can regulate the expression of neuroprotective genes, such as tyrosine hydroxylase, but also the transcription of PD-causing genes, such as UCHL1, through promoting hypomethylation. However, these inhibitors also demethylate the SNCA intron 1, producing an increase of α-syn protein expression [85]. Administration of RG-108, another DNMT inhibitor, reduces global levels of DNA methylation, exacerbating levodopa-induced dyskinesias [139].

The outcome of the HDAC inhibitors, however, is to counteract the effects of dopaminergic neurotoxin exposure (6-OHDA, MPTP or paraquat), or disease-related epigenetic changes [192]. HDAC inhibitors increase the acetylation of different histones, and consequently, increase the expression of the neuroprotective proteins, such as HSP70 -that mediate autophagy to eliminate neurotoxic proteins-, block apoptotic death induced by BCL2, or enhance the survival and morphological differentiation of dopaminergic neurons through GNDF expression [169].

There are several drugs with HDCA inhibitor activity. For instance, sodium butyrate and its derivative, phenylbutyrate, pass the blood–brain barrier and inhibit class I and II histone deacetylases with high efficiency. Additionally, both promote the expression of DJ-1, and neurotrophic factors GDNF and BDNF, protecting dopaminergic neurons from oxidative stress and α-syn toxicity. The microbial metabolites Trichostatin A and Apicidin also have HDCA inhibitory activity; in particular, Trichostatin A acts as a Class I and II HDCA inhibitor and prevents mitochondrial dysfunction, and Apicidin inhibits histone deacetylases 2 and The hydroxamate-based drug Vorinostat and Entinostat promote activation and histone di- and trimethylation, upregulating levels of HSPResveratrol is another HDAC inhibitor that activates PGC-1α via SIRT1, enhancing the levels of the mitochondrial antioxidants. Some other hyperacetylation producing drugs are Urocrotin, RGFP109, AGK2, and KHowever, Valproate acid (2-propylpentanoic acid) is the most promising drug for the treatment of PD, since it neuroprotects against rotenone-, α-syn-, lipopolysaccharide-, and MPTP-mediated toxicity through the enhancement of H3 acetylation and reduction of inflammatory mediators in microglial cells. Details about the specific epigenetic effect of these drugs and references are compiled in other reviews [191,192].

In addition to HDAC inhibition, an alternative approach is the modulation of HAT activity by HAT activators such as CTBP ((N-(4-chloro-3-trifluoromethyl-phenyl)-2-ethoxy-6-pentadecyl-benzamide) that promotes the survival and neurite growth of SH-SY5Y cells and protects them from the neurotoxin 6-OHDA [193]. An example of p300/CBP HATs inhibitors is garcinol, which protects the same cells against MPP+-induced cell death [170]. However, there has been limited research into the potential of HAT modulation as potential drug therapies for PD [194].

Selegiline and rasagiline are MAO-B inhibitors that increase the amount of dopamine in the brain, allowing the reduction of levodopa administration in PD patients (Figure 3), and at the same time contributing to the correction of reduced H3K4 methylation [140].

On the other hand, there is an increased interest in using micronutrients as a therapeutic strategy. For instance, it is known that amino acids (such as methionine and homocysteine) and vitamins (B2, B6, B12, and Folate) are involved in one-carbon metabolism affecting DNA methylation (Figure 3) [132]. Coffee drinkers present a lower risk for PD [195]. Although caffeine is thought to act as an adenosine receptor antagonist, it also seems to modulate methylation as a PDE inhibitor [154]. Vitamin E also has a protective effect by increasing MAPT promoter methylation and consequently, reducing gene expression. Coupland and collaborators showed that this micronutrient alters MAPT methylation in carriers of the H2 haplotype [111]. However, information is scarce about the direct effect of nutrient intake on genomic DNA methylation, especially regarding the combinatorial effects of nutrients with other factors, such as genetic variation and/or therapeutic drugs [132].

In general, small-molecule epigenetic modulators are able to cross the blood–brain barrier converting them into successful pharmacological modifiers of the CNS epigenetic status. However, this capacity is not shared by all the modulators, and more research on how to deliver these drugs to the brain is needed [194].

### 5.4. Potential Disease-Modifying Therapies Based on Epigenetics

In order to solve the poor blood–brain barrier penetration and off-target effects, new strategies based on regenerative treatments, such as immunotherapy, stem-cell-derived grafts, and viral gene therapy, are also being tested in several neurodegenerative disorders, including PD [184]. However, since the pathophysiology of DLB is not fully understood, the development of targeted therapeutics is challenging for this disease [183].

For PD, these therapies are designed to restore the striatal dopaminergic tone and to target the pathogenic mechanisms for disease modification. One of these mechanisms is to reduce α-syn synthesis in the striatum by silencing SNCA mRNA post-transcriptionally or reducing its translation. Another strategy could be the enhancement of α-syn clearance by increasing its intracellular degradation through autophagy-related pathways and the ubiquitin-proteasome system [184]. The different disease-modifying therapies tested for PD are described elsewhere [184,185,196,197].

DNA methylation is an attractive approach to modulate SNCA expression. In this sense, Kantor and collaborators developed a system based on epigenome editing that allows for tight downregulation of SNCA expression levels in hiPSC-derived dopaminergic neurons from a PD patient. In particular a CRISPR-deactivated Cas9 fused with the catalytic domain of DNMT3A, delivered by a lentiviral vector for targeted DNA methylation editing within intron 1 was designed [198].

However, not all clinical trials have reached satisfactory results in PD [197]. One reason could be the heterogeneity of the disease, and another, that it is often assumed that each targeted mechanism of disease applies for most patients with the same clinical diagnosis [199]. Given the many clinical phenotypes of PD, individualization of therapy and precision of treatment should be considered in the future.

In the case of DLB, the test of disease-modifying therapies is still far. DLB is often under- or misdiagnosed, and its pathology is considered to be heterogeneous since it includes neural loss, cholinergic degeneration, AD, and vascular pathology in addition to Lewy body pathology. Furthermore, Lewy bodies are found across the whole LBD spectrum, including PD and PDD. Therefore, neither genetic nor other direct biomarkers have been identified to date, and novel drug targets are needed to develop DLB-specific disease-modifying therapies [183].

## 6. Conclusions

LBD are multifactorial disorders where the disease-associated phenotype develops as a result of the complex interaction between genetic and environmental factors. Although some specific disease groups, including LRRK2-PD or GBA-PD, have been defined, even within these groups, the patients present heterogeneous phenotypes. This phenotypic heterogeneity is the result of the further modulation by epigenetic changes including both DNA methylation and histone modification.

As discussed throughout this review, numerous studies have addressed the investigation of methylation changes in either promoter regions of LBD-associated genes or throughout the whole genome. Histone modifications have been analyzed, and their effect on increasing pathogenicity of specific genes has also been shown. Although these findings add further complexity to LBD pathogenesis, they have also allowed identifying new therapeutic targets for the development of disease-modulating therapies.

## 7. Future Directions

Intense research on epigenetic modification related to LBD, especially to PD, has been carried out over the past decade, and numerous findings in the field have revealed that epigenetic modifications are associated with the modulation of the disease course. Additionally, drugs to revert disease-associated epigenetic alterations are being developed.

All studies carried out so far represent a solid base for the further systematic characterization of the dynamics of epigenetic changes in LBD. This characterization should include the analysis of brain material, cerebrospinal fluid, blood and its components, and saliva. Brain material should comprise those brain areas affected in LBD, and clinicopathological correlation should be used to differentiate between PD and DLB brains. Samples collection should be carried out at different disease stages to achieve the entire picture of disease development and progression.

The complexity of LBD arises from genetic variability and environmental factors and is further increased by methylation changes of CpG islands found in promoter regions in disease-causing as well as risk-modifying genes. However, promoter methylation might regulate the expression of individual gene transcripts affecting a very specific portion of gene products, as it has been observed for SNCA or MAPT. The role of miRNA and long noncoding RNA (lncRNAs) in gene regulation is also being studied, and various of these molecules have been involved in LBD as regulators of SNCA or GBA. At the same time, about 50% of miRNA and lncRNA genes are also regulated by methylation changes in CpG islands. An example is shown in Figure 1; the third intron of SNCB contains the miRNA gene MIR4281, and the first intron of MAPT contains a lncRNA, the MAPT intronic transcript 1 (MAPT-IT1), and both are preceded by CpG islands. Many of miRNAs or lncRNA with intra- or intergenic regions are located in the vicinity of CpG islands. The regulation of other miRNAs and lncRNAs can be either through histone modifications or the combination of both histone modification and methylation. Genetic variation within these regions may entangle the regulation machinery even more.

These observations underline that molecular diagnostic tests should not only examine for the presence of disease-causing mutations, but should include different panels to analyze common, but disease-modifying variants, epigenetic alterations, and expression levels of miRNAs and lncRNAs. The integrated analysis of these panels will then indicate which disease-modifying treatment is the most appropriate in each case. In conclusion, the establishment of personalized medicine for LBD is challenging, and although considerable advances have been made in the case of PD, for DLB, corresponding research is still beginning.
